# A Pharmacological Review of Calcitonin Gene-Related Peptide Biologics and Future Use for Chronic Pain

**DOI:** 10.7759/cureus.35109

**Published:** 2023-02-17

**Authors:** David Cooper, William D Laidig, Alexandra Sappington, Gordon MacGregor

**Affiliations:** 1 Medicine, Alabama College of Osteopathic Medicine, Mobile, USA; 2 Emergency Medicine, Alabama College of Osteopathic Medicine, Mobile, USA; 3 Pharmacology, Alabama College of Osteopathic Medicine, Dothan, USA

**Keywords:** treatment of chronic pain, chronic visceral pain, opioids, chronic pain, cgrp, calcitonin gene-related peptide

## Abstract

Calcitonin gene-related peptide (CGRP) antagonist medications have become the mainstay of acute and chronic migraine management in the outpatient setting and look to become more widely utilized by clinicians once the medications become available in generic form. However, their role in practice has remained limited to the treatment of migraines despite the ubiquitous presence of the molecule throughout the body. The literature surrounding expansion of the utility of these medications is limited; however, there have been several promising publications, and further studies are in the process to quantify their utility in the treatment of other pain-related disorders. This is a qualitative review of the current literature surrounding CGRP, particularly in relation to the treatment of non-migraine pain conditions, and looks to suggest potential utility in the field of chronic pain.

## Introduction and background

Rapid progress has been made in medicine toward the development of and treatment with biological therapies. The mainstay in biological therapy is fusion protein and monoclonal antibodies (mABs) that target certain receptors in the body, and their applications in medicine have become diverse over the last decade. Biological treatment covers many immunological dysfunctions with diverse targets such as rheumatoid arthritis, irritable bowel disease, and specific cancers [1]. Recently, a new target for monoclonal treatment has been calcitonin gene-related peptide (CGRP) and the receptor that it binds to. Currently, their use has been limited to the treatment of migraines through the blockade of CGRP receptors, and this class of drugs has been shown to be well tolerated in patients in clinical trials [2]. However, studies are ongoing to expand the potential therapeutic benefits of CGRP antagonism in patients with chronic visceral pain, trigeminal neuralgia, fibromyalgia, peripheral neuropathy, and opioid tolerance. This comprehensive pharmacological review focuses on current CGRP drugs, indications, and potential targets, as well as discusses proposed mechanisms of action related to the therapeutic benefit of CGRP antagonism.

Currently, there are two classes of drugs that inhibit the physiological action of the CGRP receptor, with the first being the gepants such as atogepant, which are immunoglobulin CGRP receptor antagonists. The second class includes the CGRP mABs, which are large molecules that act as antagonists of the CGRP receptor or bind to and inhibit the action of the CGRP ligand [3]. Usually, maintenance migraine therapy has involved certain drugs that were developed originally for diseases other than migraines, such as epilepsy, depression, and hypertension [4]. A study comparing topiramate, an epilepsy drug with an indication for the treatment of migraines, to the new mABs targeting CGRP or its receptors showed a similar efficacy in the prevention of migraines. However, mABs demonstrated a far superior safety profile and had a higher efficacy as compared to topiramate, as shown through number needed to treat (NNT) and number needed to harm (NNH) statistical models [5]. Based on the 50% responder rate (RR) and those who discontinued due to adverse effects (AEs), the NNT, NNH, and likelihood to help or harm (LHH) for the CGRP(R) mAbs were 6, 130, and 24.3:1, respectively. For topiramate, these values were 7, 9, and 1.8:1, respectively [5].

Recent research on CGRP has led to an expansion of potential target therapies that include chronic visceral pain, the enteric nervous system, ischemic cardiovascular events, opioid tolerance and withdrawal, and lung inflammation from COVID-19 infections [6-8]. Many of these treatment avenues are still undergoing clinical trials and thus further research is warranted. The future of CGRP therapy is promising in the treatment of many pathologies. Due to the ubiquitous involvement of CGRP in the human body and the already established treatment regimen for migraines, we suggest that the next logical leap for CGRP therapy may be to expand its use for the treatment of chronic visceral and somatic pain [9-11].

Currently, in the United States, there has been an ongoing opioid epidemic. It has been stated to be one of the most alarming public health crises in the history of the United States [12]. Current responses have fallen short due to its multifactorial nature that includes geographic and demographic diversity [12]. Opioids have a significant therapeutic value in analgesia and a gold standard for treatment of pain. However, the risk of addiction/dependence and the opportunity for misuse cloud the benefits of opioid therapy and create hesitancy for providers to prescribe [12]. Thus, other therapeutic approaches are needed, and CGPR-directed gepants are a logical alternative.

## Review

Pain and The Current Multimodal Approach of Treatment

Pain treatment is one of the most challenging problems that physicians deal with on a day-to-day basis. Quantifying pain is difficult due to it being subjective in nature [13]. A patient can have an identical injury to another and display a completely different presentation of symptomatology. For example, two patients with the exact degree of severity and level affected due to a spondylolisthesis can have marked variations in the degree of radiculopathy or “pain” [9]. Chronic pain is defined as somatic or visceral pain that lasts greater than three months after an injury [14]. Chronic pain may not follow dermatomal distributions that are normally associated with injury, disease, or surgical condition. Chronic pain is often seen coexisting with psychological disorders such as depression or anxiety. [12].

Currently, treatment of chronic pain is often multimodal due to the complex pathophysiology that encompasses this syndrome. Treatment may consist of a combination of medications (including opioids), physical rehabilitation, changes to lifestyle, and other interventions such as spinal cord stimulation [10]. Additionally, treatment is also aimed at the psychological manifestation of chronic pain with medications such as selective serotonin reuptake inhibitors and treatments such as cognitive behavioral therapy. This review suggests the need to consider CGRP receptor antagonists, with the hope to increase quality of life through reduction of persistent pain and reduction of overall pharmacological burden on the population with chronic pain.

Long-Term Opioid Effects

Recently, large strides have been made in the development of new analgesics for the treatment of chronic pain, but opioids are still the foundation of most pain treatment plans. Opioids are regularly prescribed for acute and chronic pain conditions, and the variation of the treatment plan is related to the length of time that opioids are prescribed [15]. Although opioids are effective analgesics, habituation is problematic, especially when these drugs are used for long durations of time. Other AEs include miosis, constipation, tolerance, and hyperalgesia.

The medical definition of tolerance is a reduction in effect following a prolonged administration of a drug that results in the loss of potency of the drug. Tolerance has been pharmacologically measured and determined to be a shift to the right in the dose-response curve [16]. Multiple mechanisms can cause tolerance in a patient, varying from genetic predispositions to uncoupling of the opioid receptors, and post-receptor adaptation. The rate of development and the extent of severity can be greatly modulated depending on the dose, frequency of administration, and drug interaction with the specific opioid receptor [17]. Tolerance is one of the most problematic factors when treating chronic pain patients, as a continual increase in opioid dose is not feasible in most cases. The FDA has defined opioid tolerance as a dose of 60 mg MME (morphine milligram equivalents) a day, and there is increased scrutiny when prescriptions over 90 mg MME per day are prescribed to patients as this increases their risk of overdose [18]. Tolerance and the mechanisms by which it occurs are not the focus of this review. Instead, we will discuss how CGRP antagonists are considered alternatives that may attenuate the effects of tolerance in long-term opioid therapy.

The other severe long-term effect of analgesic treatment is a medical paradox. Over time, it has been observed that patients' pain levels rise with opioid treatment. This has been labeled as opioid-induced hyperalgesia (OIH). OIH has been defined as a state of physiological disruption in which nociceptive sensitization has occurred due to exposure to opioid therapy [19]. OIH can present with a similar pain distribution or it may differ and present with completely altered pain presentation than the original [19]. An association was observed between heat-pain perception and varying opioid dosage in patients diagnosed with chronic pain [20]. The results revealed a correlation between increasing MME and an increase in hyperalgesia in this cohort. It was also observed that when tapering the MME, there were lower values of hyperalgesia. What can be taken from this is that the severity is variable, although it can be noted that there seems to be a strong correlation between dosage and time of these AEs. It is our hypothesis that CGRP antagonist therapy will help alleviate some of these issues in the future, which will be discussed in length in the following sections of this paper.

CGRP and Its Role in Pain

CGRP is a ligand for receptors in the G protein-coupled receptor class. It is a 37-amino acid peptide that is formed from the alternative splicing of the calcitonin/CGRP gene [21]. The CGRP receptor complex has three subunits: calcitonin-like receptor (CLR), receptor activity-modifying protein 1 (RAMP1), and receptor component protein (RCP) [22]. The receptor contains the common seven-transmembrane domain and requires RAMP1 for two mechanisms; the first is for the protein to be trafficked to the plasma membrane, and the second is for the binding to CGRP. Ultimately, the RCP facilitates coupling to Gαs (the G protein subunit that stimulates adenylyl cyclase), which initiates its second messenger cascade [23].

CGRP and its receptor have been observed in somatic and autonomic peripheral nerves, the enteric system where it has been seen to increase gastric acid secretion, and the cardiovascular system where it has been noted to increase heart rate, and has progressively been studied and described in neuroanatomical tracts of the central nervous system (CNS) [24]. CGRP has been found in specific peripheral nerves such as Aδ- and C-fibers, which has generated interest in this peptide and its specific role in pain transmission; however, at this time, its complete role in pain transmission is largely unknown [6].

CGRP has been the most recent target for therapeutic treatment of migraine. One theory believes that one of the main causes of migraines is due to a defective processing that occurs in the CNS that leads to the perception of non-noxious trigeminovascular input as pain [25]. When looking deeper into this mechanism of action, it was found that within the trigeminal nerve, the most abundant neuropeptide was CGRP, which was expressed within 35-50% of neurons in the trigeminal ganglia [25].

CGRP is expressed throughout the body and is co-localized with L-glutamate, an excitatory amino acid, in high-threshold sensory afferent fibers in the dorsal horn of the spinal cord [26]. CGRP has a well-documented role as a pronociceptive and pro-inflammatory neurotransmitter at the spinal level as it is released centrally from nociceptive fibers in response to noxious stimuli [24,26] and leads to a downstream release of several other proinflammatory and nociceptive transmitters such as substance P (SP), prostaglandins, and leukotrienes. In summary, it has been seen that CGRP expression leads to release of spinal SP, augments SP-induced nociception, and prolongs its nociceptive effect by inhibiting degradation [27,28], which amplifies further signaling to downstream effectors on ascending afferent tracts, leading to perceived pain. Research has shown that CGRP is elevated in the external jugular venous blood of patients during an acute migraine attack but not in the peripheral cubital fossa blood [29]. This has led us to appreciate the significance of CGRP as an upstream signal neuropeptide in acute inflammation and nociception.

*CGRP and Central Sensitization*

Central sensitization is a phenomenon by which, at the molecular level, the threshold to propagate action potentials is lowered and less stimuli is needed to transduce a signal to the brain. In relation to pain, we can categorize this as hyperalgesia or allodynia [11]. Hyperalgesia is a state in which a pain-inducing stimuli produce a greater than expected pain response, while allodynia is a state in which pain is experienced by a stimulus that would not normally elicit pain. CGRP has been noted to have a prominent role in this phenomenon, and we will discuss this mechanism of action here. Normally, in a pain response, a stimulus causes an action potential to be sent to the posterior gray column of the spinal cord by afferent nerve fibers [11]. Here, the primary nerve fibers synapse with second-order neurons in the dorsal horn, causing CGRP, glutamate, SP, and other neuromodulators to be released, which stimulate the second-order neurons, causing an opening of calcium and sodium channels [30]. This leads to the generation of an action potential that propagates to the thalamus through the spinothalamic tract producing the sensation of pain [31]. Continuous stimulation of the dorsal horn may result in central sensitization, leading to the sustained sensation of pain in the context of chronic pain [11]. This sensitization and sustained activation of the afferent pathway is primarily dependent on the activation of the N-methyl-d-aspartate (NMDA) receptors of the postsynaptic neurons [6]. As the primary afferent signal is transduced, it reaches a postsynaptic neuron, which activates the NMDA receptor. This receptor causes the release of nitric oxide (NO), prostaglandin E2, and glutamate, which act in a retrograde fashion to promote the further release of excitatory neurotransmitters from the nerve terminals of the primary afferents, thus maintaining a sensitized state [6,11]. It is worth noting that both an increase in excitatory transmitters and a lower threshold are required to achieve signal transduction. The release of CGRP from primary afferent fibers, which activate the CGRP receptor, will also lead to an activation of the aforementioned signal cascade and will lead to the sensitization of the NMDA receptor [31]. As the sustained signal cascade persists, phosphorylation of the NMDA receptor is triggered through the mitogen-activated protein kinase (MAPK), which leads to increased transcription factor activation [11,32]. The combined result of the pathways discussed above will lead to a state of sustained, elevated nociceptive signaling and, thus, chronic pain.

Theoretical Risks of Inhibition of CGRP

CGRP has a direct effect on vasodilation; hence, it could be hypothesized that using a biological therapy to inhibit the effect of CGRP could potentiate vasoconstriction in small arteries. However, multiple studies using different model systems, such as in vitro, dogs, and humans, have shown no significant vasoconstriction of the coronary arteries. Most importantly, there was no effect on global or regional cerebral blood flow in humans [33-35]. The study conducted by Verheggen et al. [33] showed that CGRP antagonists restored normal tonus in already dilated arteries, but no abnormal vasoconstriction was observed.

Another hypothetical concern would be that a potential vasoconstrictive effect of CGRP antagonists would compete with concurrent antihypertensive treatment and this could negate their therapeutic effects. However, it was reported in a double-blind placebo-controlled study of telcagepant given after nitroglycerin that no vasoconstrictor effect was noted [36].

There is also concern that CGRP antagonists may inhibit compensatory vasodilation during ischemia. This was addressed by a study on patients with exercise-induced stable angina who were given supratherapeutic doses of telcagepant or a placebo before they underwent exercise on a treadmill. The data showed that there were no significant differences between the two groups [37].

These theoretical adverse reactions concern cardiovascular effects of CGRP, but no significant pathology was observed for any of these. Thus, CGRP could be used as adjunctive therapy with current pharmacologic therapies for chronic pain syndromes due to their benefits greatly outweighing any potential adverse reactions.

CGRP and Opioid Tolerance

**Figure 1:**
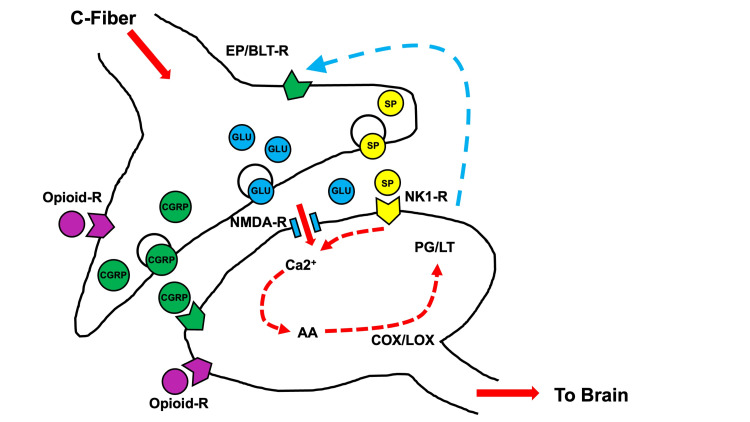
Theoretical pathway of opioid use and CGRP release in the synaptic cleft of ascending spinal neurons Binding of opioids to the opioid receptor on pre- and postsynaptic neurons is shown. Adapted from [27]. CGRP, calcitonin gene-related peptide; COX, cyclooxygenase; LOX, lipoxygenase enzymes; NMDA, N-methyl-D-aspartate; NK1-R, neurokinin-1 receptor antagonist; EP, prostaglandin E2; BLT-R, leukotriene B(4)

**Figure 2:**
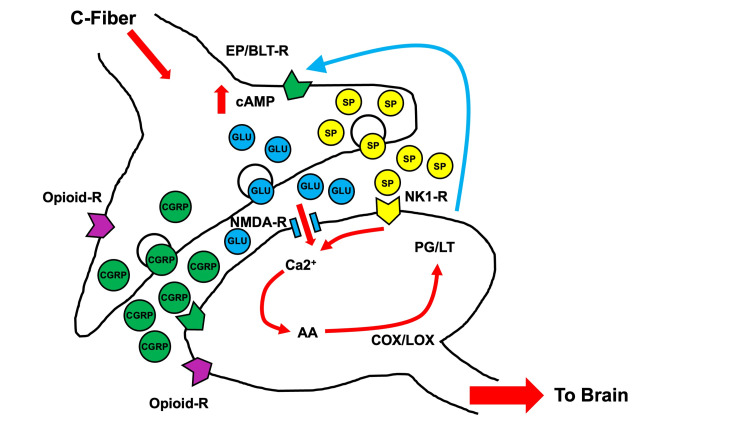
Theoretic pathways of opioid withdrawal and enhanced CGRP, L-glu, and substance-P release in the synaptic cleft of ascending spinal neurons Increased neurotransmitter release occurs after opioid withdrawal, evident in an increase in CGRP, glutamate, substance P, and secondary prostaglandins and leukotrienes. Activity is further increased by CGRP gene activation. Adapted from [27]. CGRP, calcitonin gene-related peptide; COX, cyclooxygenase; LOX, lipoxygenase enzymes; NMDA, N-methyl-D-aspartate; NK1-R, neurokinin-1 receptor antagonist; EP, prostaglandin E2; BLT-R, leukotriene B(4)

It is well known that repeated exposure to morphine and other opioid analgesics leads to tolerance and dependence, which limits their usage in the management of pain [38]. Tolerance is the loss of analgesic potency, and physical dependence results in the development of an altered physiological state that is revealed by an opioid withdrawal syndrome involving autonomic and somatic hyperactivity [27]. The molecular mechanism by which this tolerance develops has been hypothesized and is shown in Figure 1 (opioid use) and Figure 2 (opioid withdrawal). As mentioned, CGRP is pronociceptive at the spinal level and is released centrally in response to noxious stimuli [39]. It has also been demonstrated by Menard et al. [40] that the use of continuous intrathecal morphine infusions led to an increase in CGRP-like immunoreactivity in the superficial laminae of rat spinal dorsal horns [40]. In this experiment, the researchers noted that changes in spinal CGRP markers coincided with a decline in antinociception and a loss in drug potency, as reflected by an increase in morphine ED50 value [41]. These investigators also showed that increased CGRP markers and decreased drug potency were prevented by the administration of CGRP 8-37, a competitive CGRP-receptor antagonist. This suggests that overactivation of CGRP receptors in the dorsal horn contributes to the induction of opioid analgesic tolerance.

A further study proved this theory by administering CGRP 8-37 along with spinal morphine to animal subjects that were already spinal morphine tolerant and effectively reversed the increase in CGRP-like immunoreactivity and the loss of drug potency [42]. The exact cellular mechanism of this phenomenon has been similarly researched through the use of cultured adult dorsal root ganglion neurons. Studies by Ma et al. [43], showed that opioid-induced increase in CGRP can occur through activation of mu-, delta-, or kappa-opioid receptors via a protein kinase C-dependent signaling pathway [43,44]. They also demonstrated upregulation of CGRP through involvement of the MAPK pathway, which involved phosphorylation of cyclic AMP response element binding protein (CREB), a transcription factor regulating CGRP gene expression [45]. In summary, spinal morphine likely initiates gene transcription leading to increased spinal CGRP levels.

Current CGRP Drugs in Use

Several CGRP antagonists are currently on the market, and the majority of these are mABs (Table 1). Erenumab is a human immunoglobulin G2 mAB, which was originally produced from hamster ovary cells [46]. Erenumab competitively inhibits the binding of CGRP to its receptor [46]. It is dosed at 70 mg subcutaneously one to two times a month, or one dosage a month at 140 mg [47]. Erenumab has been shown to have mild adverse side effects, which ranged from pain at injection site to constipation, cramps, and muscle spasms [47]. Renal and hepatic impairments were not seen to affect the pharmacokinetics or erenumab [47]. Current contraindications for erenumab are a hypersensitivity to the drug, excipients, or latex, the latter of which are components of the drug formulation [47].

**Table 1:** Current FDA-approved CGRP medications CGRP, calcitonin gene-related peptide; SC, subcutaneously

Drug Name	Approved	Indications	Dose	Side Effects	Future Indications
Erenumab; first approved	2018	The preventive treatment of migraine in adults	70 mg SC once or twice a month, or 140 mg once a month	Injection site pain, erythema, pruritus, constipation, cramps, and muscle spasms	Trials for efficacy in temporomandibular disorder, rosacea, hot flashes, stable angina, and trigeminal neuralgia
Fremanezumab	2018	The preventive treatment of migraine in adults	225 mg SC once a month or 675 mg every 3 months	Injection site reactions such as pain, induration, and erythema	No current trials are noted for new indications
Eptinezumab	2020	Treatment or prophylaxis of migraines	Recommended dosage is 100 mg IV infusion over 30 minutes every three months	Nasopharyngitis angioedema, urticaria, facial flushing, and rash. Hypersensitivity reactions can occur days later	Trials for treatment of cluster headache syndrome are in the final stages
Galcanezumab	2018	Treatment of prophylaxis of migraines. Treatment of cluster headaches	A loading dose of 240 mg SC. Followed by 120 mg monthly maintenance dose	Injection site reactions such as pain, erythema, induration, and pruritus. Hypersensitivity reactions are uncommon but have been noted	No current trials are noted for new indications
CGRP antagonists “gepants”					
Rimegepant	2020	Acute treatment of migraine with or without aura in adults	Oral dissolvable tablet 75 mg lingual or sublingual	Nausea and rare hypersensitivity reactions with associated dyspnea and rash	Trials for treatment of trigeminal neuralgia are in the final stages
Ubrogepant	2019	Acute treatment of migraines with or without aura	Oral dose or 50 mg or 100 mg. May take another dose after 2 hours. Should not exceed 200 mg in 24 hours	Sedations, somnolence, and dryness of mouth were noted. Increased incidence was noted with increased dose	Trials for indication for cluster headaches may be researched in the future
Atogepant	2021	The preventive treatment of episodic migraine in adults	Once a day oral tablet that is dosed at either 10, 30, or 60 mg	Constipation, nausea, somnolence, decreased appetite, and weight loss	Trial for weight loss may be researched further

Eptinezumab is a humanized immunoglobulin G1, which selectively binds to the α and β forms of the CGRP ligand and inhibits the activation of the receptor [48]. The administration of this drug is through an intravenous infusion that is dosed at 100 mg every three months [48]. Eptinezumab has shown AEs such as nasopharyngitis angioedema, urticaria, facial flushing, and rash [48]. Hypersensitivity reactions have also been seen to occur days after the drug has been administered [48]. The only contraindication at this time is patients who suffer from hypersensitivity reactions [48].

Galcanezumab is also a member of CGRP-directed drugs. It is a humanized IgG4 mAB that was created from hamster ovaries [49]. Like the previous mABs, it selectively binds and inhibits CGRP-induced receptor activation. Unlike the other two previously mentioned drugs, it also inhibits capsaicin-induced vasodilation, which allows for prolonged relaxation of the coronary arteries [49]. It is a subcutaneous injection that is dosed as consecutive injections of 120 mg each as loading doses, followed by 120 mg injection once a month [49]. Adverse reactions were similar in that injection site pain was noted, as well as hypersensitivity reactions days after administration [49]. It carries the contraindication of patients who suffer from hypersensitivity reactions. Other notable mABs are fremanezumab and the gepants class of drugs, which includes rimegepant, ubrogepant, and atogepant.

Discussion on Future Benefits of CGRP Usage

Potential new uses for CGRP antagonists are now being investigated (Table 1). The first of these is trigeminal neuralgia. Trigeminal neuralgia is a common cause of neuropathic facial pain in the elderly population. This disease state has been described as a recurrent, very severe stabbing pain that is localized around the distribution of the trigeminal nerve. It is commonly triggered by stimuli such as talking [50]. A recent study showed that blood samples taken from 20 individuals with the diagnosis of trigeminal neuralgia revealed significantly elevated levels of CGRP when compared to controls [51]. Recent clinical studies have led researchers to believe that the CGRP pathway may be involved in trigeminal neuralgia [50]. Several ongoing studies are presently assessing the safety and efficacy of CGRP antagonism on refractory cases of trigeminal neuralgia [52,53].

Another proposed use of CGRP antagonists is in the treatment of peripheral neuropathy. The specific subtypes that have received research efforts are diabetic neuropathy and chemotherapy-induced neuropathy. The mechanism of the pathophysiology of diabetic neuropathy is currently up for debate, but recent theories point to an increase in oxidative stress in dorsal root ganglion neurons caused by hyperglycemia, leading to mitochondrial dysfunction [54]. One study of interest investigated the therapeutic benefits of a microneedle patch delivering CGRP antagonist for the treatment of localized neuropathic pain in rat subjects [55]. The study used several neuropathic pain models including diabetic neuropathy, spared-nerve injury, and ultraviolet B radiation-induced neuropathy. It was found that the patches produced effective analgesia on neuropathic pain without disturbing the normal nociception and motor function [55]. The pathophysiology for chemotherapy-induced neuropathy seems to be multifactorial and involves oxidative stress, apoptotic mechanisms, altered calcium homeostasis, axon degeneration, and membrane remodeling, as well as immune processes and neuroinflammation [56].

In another study, 3-hydroxyflavone (3-HF) was used to attenuate nociception and paclitaxel-induced neuropathic pain in mice models through the downregulation of mRNA expression of several inflammatory cytokines including tumor necrosis factor-α (TNF-α), interleukin-1β (IL-1β) and interleukin-6 (IL-6), CGRP, and SP [57]. Paclitaxel is a common chemotherapeutic agent that inhibits microtubules and commonly causes peripheral neuropathy in patients. It was found that 3-HF, through mechanisms of downregulation of CGRP levels and other secondary signaling neuropeptides, alleviated the nociceptive pain, paw edema, development of tactile and cold allodynia, and hyperalgesia of the subjects. While more research is indicated to gain data regarding treatment efficacy, the current evidence suggests a promising future for CGRP therapy in the treatment of neuropathy.

Fibromyalgia is a commonly diagnosed pain disorder, but is one that is very difficult to treat. Fibromyalgia has been described as variable and diffuse hyperalgesia, fatigue, weakness, psychiatric symptoms, and somatic disorders [58,59]. The underlying pathology of fibromyalgia is still not well understood, but the literature points toward a focal disturbance involved in the central neural processing in nociceptive pathways [60]. As discussed in previous sections, CGRP is directly involved in hyperalgesia, and targeting the receptors through delivery mechanisms such as epidural injections could potentially be therapeutic to patients suffering from fibromyalgia.

Temporomandibular disorders (TMDs) are another set of disorders that have been linked to abnormal physiology of CGRP. TMDs are described as pain stemming from the temporomandibular joint and muscles related to mastication [50]. There have been previous studies that have documented a connection between TMDs and migraines [50]. Due to this connection, there is a strong assumption that CGRP may be a suitable target for TMD therapy [50].

## Conclusions

The future of pharmacological treatment is shifting toward biological therapy. The efficacy of biological therapy and side effect profile has been attractive and has promoted research and funding into this area of medicine. The CGRP targets show promise in the treatment for more than just migraine therapy; however, much more research is needed to be conducted for other potential therapeutic targets. The mechanism of action of CGRP antagonists has been detailed in this review, and we suggest that these new drugs may have the potential to be an effective alternative to opioid treatment for patients with chronic pain. Our goal was to inform readers on this drug class, how CGRP works physiologically, and the potential for alternative treatment of chronic pain. Much remains to be discovered about CGRP, and future discoveries will certainly benefit patients who suffer from chronic pain.
